# Newer Horizon of Mesenchymal Stem Cell–Based Therapy in the Management of SARS-CoV-2–Associated Mucormycosis: A Safe Hope for Future Medicine

**DOI:** 10.3389/fmicb.2021.738983

**Published:** 2021-10-11

**Authors:** Alok Raghav, Syed Ghazanfar Ali, Goo-Bo Jeong, Kirti Amresh Gautam, Shahid Banday, Qazi Noorul Mateen, Prashant Tripathi, Richa Giri, Saurabh Agarwal, Manish Singh, Haris M. Khan

**Affiliations:** ^1^Multidiscplinary Research Unit, Department of Health Research, MoHFW, GSVM Medical College, Kanpur, India; ^2^Viral Research Diagnostic Laboratory, Department of Microbiology, Jawaharlal Nehru Medical College and Hospital, Aligarh Muslim University, Aligarh, India; ^3^Department of Anatomy and Cell Biology, College of Medicine, Gachon University, Getbeol-ro Yeonsu-gu, Incheon, Korea; ^4^Department of Molecular, Cell and Cancer Biology, University of Massachusetts Medical School, Worcester, MA, United States; ^5^Department of Biochemical Engineering and Biotechnology, Indian Institute of Technology Delhi, New Delhi, India; ^6^Department of Biochemistry, GSVM Medical College, Kanpur, India; ^7^Department of Medicine, GSVM Medical College, Kanpur, India; ^8^Department of Neurosciences, GSVM Medical College, Kanpur, India

**Keywords:** mesenchymal stem cells, Mucormycosis, SARS-CoV-2, COVID-19, immunomodulation

## Abstract

SARS-CoV-2–infected patients are reported to show immunocompromised behavior that gives rise to a wide variety of complications due to impaired innate immune response, cytokine storm, and thrombo-inflammation. Prolonged use of steroids, diabetes mellitus, and diabetic ketoacidosis (DKA) are some of the factors responsible for the growth of Mucorales in such immunocompromised patients and, thus, can lead to a life-threatening condition referred to as mucormycosis. Therefore, an early diagnosis and cell-based management cosis is the need of the hour to help affected patients overcome this severe condition. In addition, extended exposure to antifungal drugs/therapeutics is found to initiate hormonal and neurological complications. More recently, mesenchymal stem cells (MSCs) have been used to exhibit immunomodulatory function and proven to be beneficial in a clinical cell-based regenerative approach. The immunomodulation ability of MSCs in mucormycosis patient boosts the immunity by the release of chemotactic proteins. MSC-based therapy in mucormycosis along with the combination of short-term antifungal drugs can be utilized as a prospective approach for mucormycosis treatment with promising outcomes. However, preclinical and in mucormyIn mucormycosis, the hyphae of clinical trials are needed to establish the precise mechanism of MSCs in mucormycosis treatment.

## Introduction

**Figure 1:**
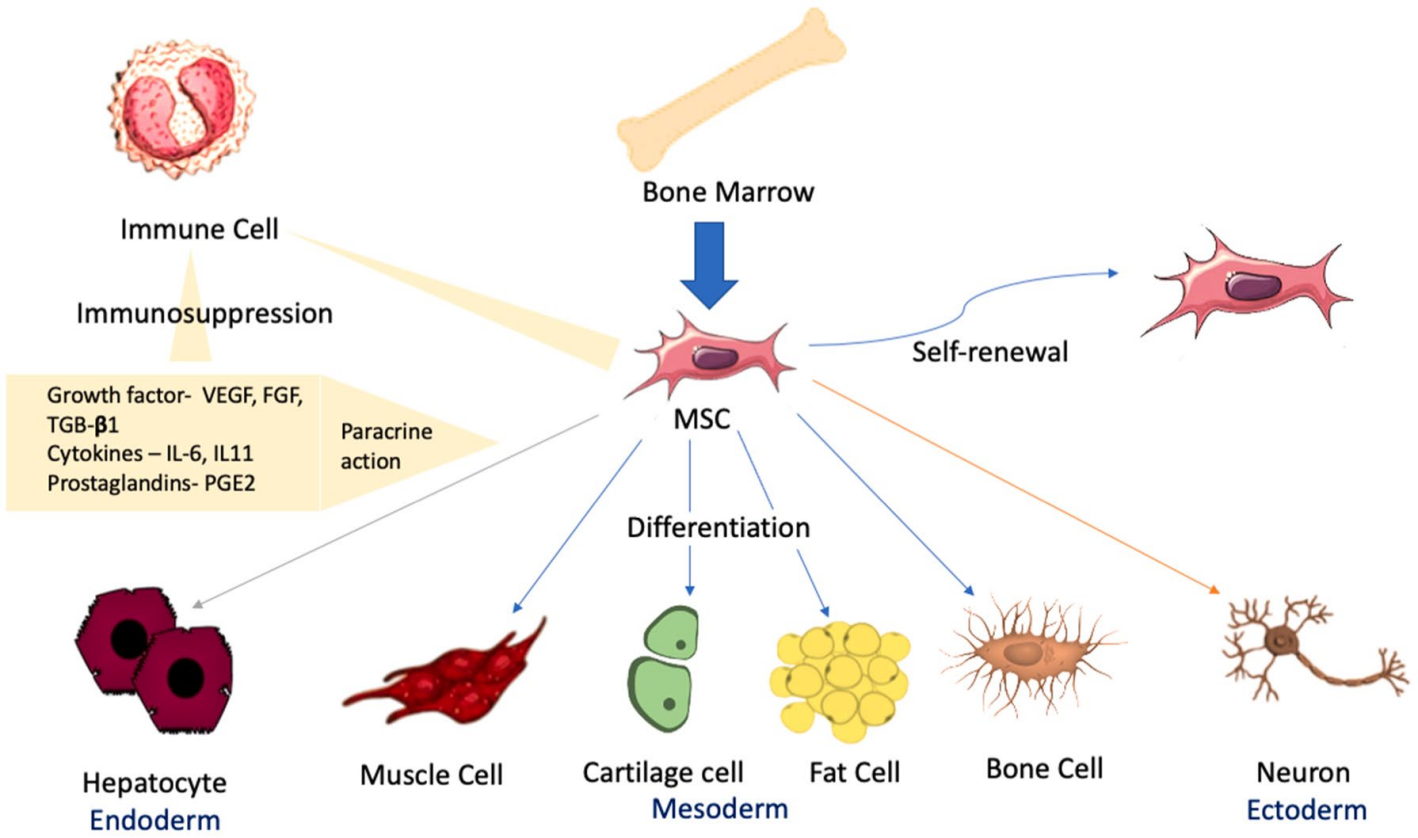
The differentiation of MSCs into various organs and tissues.

Mesenchymal stromal cells (MSCs) are multipotent adult stem cells that exhibit their presence in several tissues, including bone marrow, umbilical cord, and fat tissues. These cells differentiate into multiple tissues, such as bone, cartilage, connective tissue, muscle, and fat cells (Krampera, 2011). The International Society of Cellular Therapy (ISCT) defines MSCs as cells that (i) should be plastic adherent in defined standard culture medium and conditions; (ii) tend to differentiate *in vitro* into adipocytes, osteoblasts, and chondroblasts; and last (iii) should express immunophenotypic markers, including CD73, CD90, and CD105 except for CD14, CD34, CD45, and class II major histocompatibility complex molecules (Dominici et al., 2006; Figure 1). MSCs play a significant role in regulating T cell subsets, B cells, natural killer (NK) cells, neutrophils, and monocyte-derived cells as these exhibit the characteristics to interact with the immune system (Bernardo and Fibbe, 2013). MSCs are also involved in imparting tissue repair and regeneration (Li et al., 2021; Raghav et al., 2021). MSCs are currently being explored as a novel therapeutic approach in various clinical settings. MSC transplantation is known to provide a regenerative effect and tissue repair in several organ systems, including the central nervous system (CNS), heart, skin, and bone (Hashemian et al., 2021; Liu and Holmes, 2021).

MSCs exhibit immunomodulatory function and prove to be beneficial in clinical cell-based regenerative approaches. The U.S. Food and Drug Administration (FDA) conducted 950 successful clinical trials over 10,000 subjects performing promising MSC therapies (Pittenger et al., 2019). MSCs possess immunosuppressive characteristics and can be effectively used for the treatment of autoimmune diseases (Li et al., 2021). Moreover, several clinical results reflect a promising effect of MSCs in various diseases; however, the antimicrobial properties of MSCs are still a concern of research in clinical settings. Published research has found that transplantation of hematopoietic stem cells (HSCs) possessing immunosuppressive capabilities shows inhibition of antimicrobial immune responses that report increased risk of infection in immunocompromised individuals (Nauta and Fibbe, 2007). In another study conducted on MSCs, it is demonstrated that MSCs mediate immunosuppression in the fungal infected subject (Le Blanc et al., 2008). In a related study, it is found that the gamma/delta T cells produce IL-17, which exhibits an antifungal effect (Caccamo et al., 2011). This antifungal mechanism contributes to increased expression of Th17 levels through the infusion of IL 17+ MSCs in mice infected with *C. albicans* (Caccamo et al., 2011).

**Figure 2:**
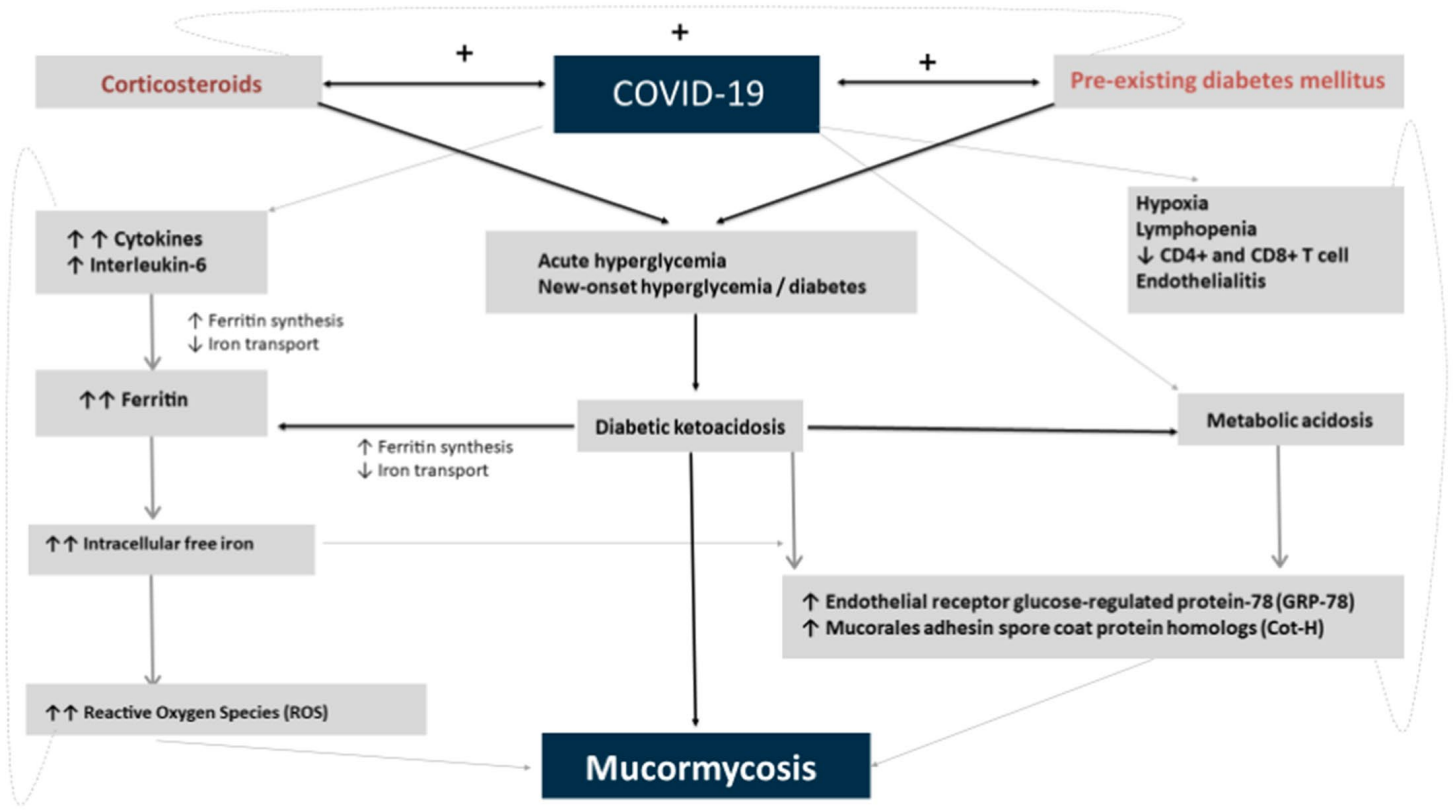
Postulated interaction of diabetes, corticosteroid, and COVID-19 with mucormycosis.

In SARS-CoV-2-recovered subjects, post COVID-19 sepsis is among the common complications. The post-COVID-19 irregularities include impaired innate immune response, cytokine storm, thrombo-inflammation, and eventual immune exhaustion. Mucormycosis contributes to life-threatening fungal infections with higher than 50% mortality rates even postsurgical debridement and after antifungal drugs (Johnson et al., 2021). Recently, several cases of mucormycosis in patients infected with SARS-CoV-2 have been reported worldwide with significant numbers in India. The leading causes that facilitate Mucorales spores to germinate in SARS-CoV-2–infected patients include hypoxia, hyperglycemia (new-onset, steroid-induced, and diabetes mellitus or DM), low pH (diabetic ketoacidosis or DKA and metabolic acidosis), increased iron levels, and impaired phagocytic activities of white blood cells contributed by immunosuppression (steroid mediated; Figure 2). These factors, along with prolonged hospitalization with and without mechanical ventilators, contribute to the significant increase in mucormycosis cases.

In around 70% of mucormycosis cases, *rhizopusarrhizus* is the culprit fungus responsible for causing pulmonary, gastric, cutaneous, and rhino-orbital-cerebral diseases, especially in adults (Johnson et al., 2021). The high pathogenicity of this fungus contributes to rapid tissue obliteration and invasion throughout tissue planes (Ibrahim and Kontoyiannis, 2013). These SARS-CoV-2–recovered patients are at higher risk of developing mold infections, such as mucormycosis, due to the application of prolonged corticosteroids and anti-IL-6–directed approaches. The present review is focused on the therapeutic aspect of MSCs in these mold-infected patients. Moreover, the pathogenesis, diagnosis, clinical features, and management of mucormycosis is also described in detail.

## Sars-Cov-2–Associated Comorbidities

The disease pattern in SARS-CoV-2–infected patients ranges from mild to severe bacterial- and fungal-associated pneumonia (Farnoosh et al., 2020). Several associated comorbidities (DM, chronic obstructive pulmonary disease) and immunocompromised conditions (prolonged corticosteroid therapy, period of stay in intensive care unit, ventilation) put the patient’s life at risk of developing opportunistic infections. Recently, several opportunistic infections, including oropharyngeal candidiasis, pulmonary aspergillosis, pneumocystis jiroveci, and bloodstream candida infections are seen in patients infected with SARS-CoV-2 (Chowdhary et al., 2020; Salehi et al., 2020). Rhino-orbital-cerebral-mucormycosis (ROCM) refers to the sino-nasal, rhino-orbital, and rhino-orbital-cerebral disease, among the most common in current clinical practices worldwide. A few cases have been reported recently in patients infected with SARS-CoV-2 (Mehta and Pandey, 2020; Mekonnen et al., 2020). In a similar trend, six cases of ROCM were also reported recently in COVID-19 disease (Sen et al., 2021). Among these six cases, one patient had concurrent COVID-19 along with mucormycosis at the time of hospitalization, and the other five patients who were given systemic steroid to manage COVID-19 developed mucormycosis (Sen et al., 2021).

In another study, 10 confirmed, clinically diagnosed orbital mucormycosis cases concurrently occurring with COVID-19 disease were reported (Sarkar et al., 2021). It is stated that the long-term use of steroids and monoclonal antibodies along with broad-spectrum antibiotics for the management of SARS-CoV-2 increases the risk of developing mold-related infections. COVID-19 infection has a proclivity for causing severe pulmonary complications with subsequent alveolo-interstitial pathology. This whole cascade itself is responsible to predispose the SARS-CoV-2–infected patient toward the development of invasive fungal infections, especially in the sinuses and lungs (Gangneux et al., 2020). A recent study demonstrates that immune dysregulation exhibits a low population of T cells, CD4, and CD8 cells (Sen et al., 2021).

It is evident that mucormycosis is an extremely rare inhabitant in healthy individuals, but in immunocompromised conditions, the patient becomes predisposed to the infection as in mucormycosis. The several predisposing complications include DM, with or without DKA, malignancies, long-term neutropenia, organ transplantation, patients of immunosuppressive drugs or corticosteroid therapy, patients with high iron intake or hemochromatosis, severe burn injuries, deferoxamine therapies, immunodeficiency syndromes, malnutrition, and open wounds (Singh et al., 2021). In a recent study, it is quoted that a cumulative dose of prednisone (>600mg) or cumulative methylprednisone (2–7g) given to the patient for a month before predisposes immunocompromised patients to develop mucormycosis (Singh et al., 2021). Another case reports patients receiving 5–14days of steroid therapies, particularly people with DM developed mucormycosis infection (Singh et al., 2021).

Mucormycosis can be present in the nose, sinuses, lungs (pulmonary), orbit, CNS, skin, bones, kidney, joints, gastrointestinal tract, and mediastinum (invasive type). Studies show that giant cell invasion, eosinophilic necrosis, and thrombosis are the prime hallmarks of mucormycosis (Singh et al., 2021). These mucormycosis-related findings in the COVID-19 pandemic offer to exploit a new horizon of the research domain, especially when there are excessive corticosteroids used in the treatment and management of COVID-19–associated complications, especially in India.

## Pathogenesis of Mucormycosis

Mucormycosis is caused by the thermotolerant saprophytic fungi named Mucorales, an inhabitant of decaying organic matter and soil (Prakash et al., 2016, 2020). In an Indian study conducted on soil, several pathogenic species, such as *Rhizopus*, *Lichtheimia*, *Cunninghamella*, *Rhizomucor*, and *Apophysomyces* are reported (Prakash et al., 2016). In another hospital-based aeromycological study, pathogenic *Mucorales* is isolated from hospital air samples (Prakash et al., 2020). It is well documented that 11 genera and 27 species are known to cause mucormycosis (Prakash and Chakrabarti, 2019). In India, *Rhizopus arrhizus* is the most common cause of mucormycosis with some contribution from *Rhizopusmicrospores* and *Rhizopushomothallicus (*Prakash and Chakrabarti, 2021*)*. ROCM is widely caused by the *Rhizopus* species and *Apophysomyces variabilis* because of their abundance in soil and air (Prakash and Chakrabarti, 2019). Some other Mucorales causative of mucormycosis in India are *Rhizomucorpusillus*, *Cunninghamella* species, *Mucor* species, *Syncephalastrum* species, and *Saksenaea* species (Frater et al., 2001; Prakash and Chakrabarti, 2021).

Patients lacking phagocytes or having impaired functional phagocytes are at more risk of developing mucormycosis (Sugar, 2005). In normal conditions, the host mononuclear and polymorphonuclear phagocytes inhibit the growth of Mucorales through the generation of oxidative metabolites, defensins, and cationic peptides (Ibrahim et al., 2012). In a previously published study, it is found that host neutrophils inhibit the growth of *Rhizopus arrhizus* as a result of which there is upregulation of toll-like receptors 2 (TLR-2) that induces the NF-κβ pathway-related genes (Ibrahim et al., 2012). In the chronic hyperglycemic environment, especially in DM and also in the case of low pH, especially DKA, the host’s phagocytes become nonfunctional and thereby do not show a protective effect (Ibrahim et al., 2012). The precise mechanism or nonfunctionality of phagocytes in DM, ketoacidosis, and corticosteroid treatment is yet to be studied in detail.

Mucorales causing mucormycosis possess virulence features that cause the disease in the host. In addition, utilizing the host’s iron is among the inhabitant traits of these Mucorales. Iron is a vital component of cell growth and development; thus, these Mucorales utilize it from the host’s cells for their growth and multiplication. In a previously published report, a free form of iron is utilized by Mucorales, causing disease in the host, thereby suggesting that bound forms of iron in proteins, such as ferritin, transferrin, and lactoferrin, are untouched (Boelaert et al., 1993; Andrianaki et al., 2018). In patients with DKA, it can be easily explained that elevated levels of free iron in their serum facilitates the growth of *Rhizopus arrhizus* at acidic pH (7.3–6.88) but not at alkaline pH (7.78–8.38; Ibrahim et al., 2012). In another study, it is observed that patients on dialysis undergoing treatment with deferoxamine (iron-chelator) predispose them to lethal mucormycosis (Boelaert et al., 1991; Corzo-León et al., 2018).

## Diagnosis of Mucormycosis

### Clinical Diagnosis

Diagnosis of mucormycosis requires a high degree of suspicion, host factor identification, and swift assessment of clinical findings. Rhino-cerebral, pulmonary, and soft tissues are the common targets of Mucorales infection. In a pioneer study, a proposed algorithm was fabricated to diagnose rhino-cerebral mucormycosis in diabetic subjects (Corzo-León et al., 2018).

Mucormycosis can also be diagnosed by radiological intervention if the multiple nodules (≥10) and pleural effusions are presentable. In computerized tomography (CT) scanning, a reverse halo sign (RHS) confirms the presence of mucormycosis (Legouge et al., 2014; Jung et al., 2015). In another study, positron emission tomography–computed tomography (PET/CT) using [18F]-fluorodeoxyglucose (FDG) was used to diagnose mucormycosis (Liu et al., 2013).

### Microscopic Examination and Culture-Based Diagnosis

Mucormycosis diagnosis using microscopic examination and cultures are a conventional and keystone approach. The implication of direct microscopy using blankophor and calcufluor while in clinical specimens rapidly diagnose the presence of mucormycosis infection (Frater et al., 2001; Lass-Flörl et al., 2007; Lass-Flörl, 2009). Mucorales nonseptate or pauci-septate hyphae (having a width of 6–25μm) shows irregular distribution with a ribbon-like feature and appearance in the microscopy. Microscopic diagnosis of fungal elements can be readily speculated using hematoxylin and eosin stains. In invasive mucormycosis, tissue histopathology exhibits infarcts and angio-invasion. In another antibody-based detection approach, mouse monoclonal anti-*Rhizomucor* antibody is used for immunohistochemical analysis to diagnose Mucorales and Entomophtorales (Lackner et al., 2014).

### Molecular-Based Diagnosis

Molecular-based diagnosis of the mucormycosis is based on the conventional polymerase chain reaction (PCR), DNA sequencing, melt curve analysis of PCR products and restriction fragment length polymorphism analyses (RFLP; Hsiao et al., 2005; Larché et al., 2005; Nagao et al., 2005; Machouart et al., 2006; Kasai et al., 2008; Nyilasi et al., 2008; Springer et al., 2016). These techniques can either be used for the detection and identification of Mucorales; 18s and 28S rRNA along with internal transcribed spacer (ITS) gene detection is the gold standard of all molecular assays in mucormycosis (Lackner et al., 2014). Studies in the past used formalin-fixed, paraffin-embedded, or fresh tissue specimens for performing the molecular assays, yet their sensitivity and specificity is variable (Lackner et al., 2014). The matrix-assisted laser desorption ionization-time of flight mass spectrometry (MALDI-TOF MS) approach is another pioneering technique for identifying Mucorales (Millon et al., 2019). Moreover, several molecular techniques, such as the FTR1 gene, cytochrome b, are used for the detection of Mucorales (Millon et al., 2019). Multiplex real-time quantitative PCR (mqPCR) using targets for the ITS1/ITS2 region of the gene with specific probes for R. *oryzae*, R. *microsporus*, and *Mucor* spp. using specific customized primers are able to detect the Mucorales species (Millon et al., 2019).

## Management of Mucormycosis

The efficient management of mucormycosis depends on mainly early diagnosis, limiting the predisposing risk factors, surgical debridement (if applicable), and implication of antifungal agents. In early management of mucormycosis, polyene therapy within 5days of early diagnosis shows significant improvement in survival compared with the therapy of polyene administered after 6days of the diagnosis (83% vs. 49% survival; Chamilos et al., 2005). Early diagnosis is credited with the invention of new molecular biology assays, including real-time quantitative PCR, that help extensively in the timely diagnosis and management of mucormycosis. In a previously published study, mqRTPCR was used against the target for the 28SrRNA gene for the diagnosis of mucormycosis by identification of *Rhizopus*, *Mucor*, and *Cunninghamella* species at the same time in a single specimen (Kasai et al., 2008). In mucormycosis-associated ROCS, CT scans exclusively present the sinusitis, ignoring the deeper infection that is also probably suggestive of mucormycosis. To overcome this issue, magnetic resonance imagining (MRI) seems to be more sensitive than CT scans as it diagnoses the orbital and CNS involvement.

In another approach for management of mucormycosis, limiting or reversal of the causes for predisposition must be the point of focus in such patients. Moreover, it is cumbersome to reverse or limit the underlying causes in such diseased patients when managing for mucormycosis infection. It is suggested to use low-dose administration for corticosteroid (immunosuppressive medication) if possible in such patients. Such an approach also includes aggressive glycemic control and normalization of the acid–base environment to prevent DKA.

Surgical management in mucormycosis involves debridement of the necrotic tissues for complete elimination of mucormycosis. This approach is the last option to treat such infected patients when there is no or limited penetration of antifungal agents/drugs at the site of infection due to tissue necrosis and blood vessel thrombosis. In a previously published study, surgical debridement of the tissue was found to be on top when tested in a logistic regression model in patients with mucormycosis with promising outcomes (Roden et al., 2005). In another study, patients who did not undergo surgical debridement showed significantly higher mortality compared with those who underwent surgical debridement of the tissue in mucormycosis disease (Asai et al., 2003).

Primary antifungal therapy includes the use of amphotericin B, liposomal amphotericin B, and amphotericin B lipid complex. The dose of these antifungal agents in the treatment of mucormycosis is still not known or fixed under treatment guidelines. However, starting dosages of 1mg/kg/day of amphotericin B and 5–7.5mg/kg/day for liposomal amphotericin B and amphotericin B lipid complex are commonly prescribed in clinical practices in both children and adults. A previously conducted study proved that a higher dose of liposomal amphotericin B (>10mg/kg/day) does not show any significant pharmacokinetic advantage in patients with mucormycosis (Walsh et al., 2001).

Salvage therapy provides an additional advantage in the management of mucormycosis. Deferasirox or posaconazole are some of the offered choices for patients with mucormycosis and those who are intolerant to polyene therapy. Sufficient data for posaconazole is available from clinical trials regarding the efficacy and safety of this drug in the management of mucormycosis. However, if deferasirox must be added, it should be given only for 2–4weeks during the salvage therapy as, beyond 4weeks, it causes toxicity (Nick et al., 2002). For patients with mucormycosis who are undergoing immunosuppressive medications, the secondary antifungal agents should be continued simultaneously. Posaconazole can be the second immediate choice of treatment if polyenes cannot be administered in such patients for a prolonged period.

In the treatment of mucormycosis, antifungal drugs are the first-line treatment options, especially in prophylaxis and invasive fungal infections. These azoles have associated long-term side effects, especially in patients with hematologic malignancies. The long-term complications or side effects associated with the azole implication include hormone-related impairments, such as alopecia, impotence, oligospermia, decreased libido, gynecomastia, hyponatremia, hypokalemia, and rarely adrenal insufficiency, along with hepatotoxicity (Benitez and Carver, 2019). In another study, it is found that voriconazole and posaconazole are causative factors for peripheral neuropathies and pancreatitis if used in combination with itraconazole (Benitez and Carver, 2019). It is also observed that voriconazole is associated with the development of complications such as periostitis, squamous cell carcinoma, and phototoxic reactions (Benitez and Carver, 2019). Because these azole therapies cause several long-term complications if used for a long time period, to overcome these associated limitations with azoles and associated complications, MSC-based therapeutic approaches could prove to be helpful, safer, and efficient without any conflict with patient’s health.

## Msc-Based Management of Covid-19–Associated Mucormycosis

Multipotent stem cells exhibit immunomodulation characteristics that were found to be safe for the treatment of COVID-19 (Akbari and Rezaie, 2020; Kumar, 2020; Wu et al., 2020). The FDA has already given its consent for using MSC-based therapy to treat COVID-19 and its associated complications. Regenerative medicine has again proven effective and efficient in curing this worldwide pandemic of COVID-19 and its associated comorbidities without any conflict. In a previously published study, IL-17, produced by MSCs, activates the NFκB pathway to downregulate TGF-β production in MSCs, resulting in abolishment of MSC-based immunomodulation (Yang et al., 2013). Moreover, these IL-17+ MSCs possess anti-Candida albicans growth effects *in vitro* and therapeutic effect in *C. albicans*–infected mice.

Mucormycosis is a life-threatening opportunistic infection that severely affects immunocompromised patients, especially SARS-CoV-2-infected patients. In a recently published case report, it is found that a patient with acute myeloid leukemia (AML) presented with pulmonary mucormycosis and was treated with the antifungal agent voriconazole preceded by a 1month transplant with peripheral blood stem cells followed by isavuconazole, and the patient showed a significant reduction in ground glass opacities in the CT scan (Aqsa and Chow, 2019). This study is suggestive of using antifungal agents in combination with stem cell transplantation for obtaining a better outcome. MSCs have a tendency to reduce inflammation, repair tissue, and do pathogenic clearance (Hashmi et al., 2016).

COVID-19 and its associated mucormycosis impair the immune system by initiating the cytokine storm in such patients. MSC-based therapy is coming up with new hope for promoting endogenous repair and inhibiting the cytokine storm, hence, helpful in managing COVID-19 and its associated mucormycosis (Rahimkhoei et al., 2021). In a previously conducted study on COVID-19 patients, injection of MSCs to lungs in pulmonary disease showed a protective effect on the endothelial cells (Liang et al., 2020). In another study published recently, it is quoted that MSC transplantation significantly reduced neutrophil-mediated inflammation of the airways by inhibiting the Th17 signaling pathway in a mouse model of asthma triggered by the fungus *Aspergillus fumigatus* hyphal extract (Arango et al., 2018). Bone marrow–derived MSCs show an immunomodulatory effect on macrophages stimulated by *Aspergillus fumigatus* conidia that further lowers the expression of tumor necrosis factor-α (TNF-α) and increases the expression of IL-10 (Arango et al., 2018).

## Host Immune Dysregulation in Mucormycosis

Bronchial alveolar macrophages (BAM) contribute as vital components of the innate immune system, assuming the first line of defense (Aberdein et al., 2013). In a murine study, it is found that BAM is unable to inhibit the growth of *R. oryzae* in an immunosuppressive environment (Waldorf et al., 1984). In another study, *Rhizopus* spp. exhibit inhibition of phagosome maturation in the presence of melanin on their spores and mediate iron metabolism that helps in regulation of immune defense (Ghuman and Voelz, 2017). Mucorales recognize the epithelial cells when they come in contact for causing infection. Epithelial cells are the first line of defense present in the outer surface of the skin and alveoli that protects against fungal pathogens (Ghuman and Voelz, 2017).

In a previously published molecular study, human epithelial cells (A-549) were observed to interact *with L. corymbifera*, *R. oryzae*, *R. delemar*, and *C. bertholletiae* mediated through platelet-derived growth factor receptor B (PDGFRB) signaling in the infection phase (Chibucos et al., 2017). Moreover, another transcriptome analysis on epithelial cells encountered with *R. arrhizus* var. *delemar* shows increased expression of epidermal growth factor receptor (EGFR) in lungs (Watkins et al., 2018). Polymorphonuclear leukocytes (PMNs) or neutrophil granulocytes play a vital role in inhibiting pathogen growth by initiating chemotactic factors such as cytokines. During mucormycosis, these chemotactic factors modulate the immune response in the infected host (Chinn and Diamond, 1982).

In a published study, it is found that there is increased generation of superoxide anion (O2^−^) when encountered with the hyphae of mucoralean species that, in turn, modulate the neutrophils (Chamilos et al., 2008b). In mucormycosis, the hyphae of *Rhizopus* spp. contribute to the activation of TLR-2, IL-1B, and TNF-α of neutrophils (Chamilos et al., 2008a). In another study, if TLRs in combination with liposomal amphotericin B is given to a Mucorales-infected host, neutrophils reduce the proinflammatory responses by switching TLR-2 to TLR-4, in turn, providing protection against Mucorales infection without any cytotoxicity (Bellocchio et al., 2005).

**Figure 3:**
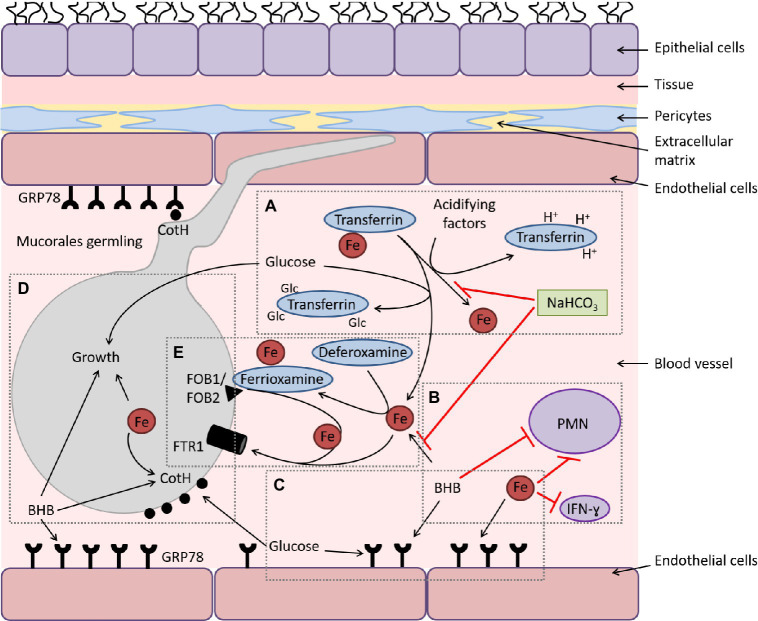
Mucorales interaction with endothelial cells during hematogenous dissemination/organ seeding and the effect of host factors on these interactions and on the immune response. **(A)** Hyperglycemia and ketoacidosis result in liberation of iron from serum-sequestering proteins (e.g., transferrin) *via* glycosylation and protonation, respectively. **(B)** Ketone bodies (e.g., BHB) and free iron negatively affect the immune response to the infection, and sodium bicarbonate (NaHCO_3_) reverses this negative effect by preventing iron release from transferrin and neutralizing acidity. **(C)** Surface expression of glucose-regulator protein 78 (GRP78) on endothelial cells is enhanced to cope with the stress elicited by hyperglycemia, free iron, and ketone bodies. **(D)** Glucose, free iron (transported by the high-affinity iron permease [Ftr1p]), and BHB also enhance the expression of the fungal cell surface CotH, which results in invasion of the endothelium and augmentation of fungal growth. **(E)** In deferoxamine-treated hosts, the iron-rich ferrioxamine binds to its fungal receptor (ferrioxamine binding proteins [Fob1/Fob2]) then releases iron *via* a reductive step prior to feeding invading *Mucorales via* Ftr1p transportation. (Adapted from Ref (Baldin and Ibrahim, 2017) under the terms of the Creative Commons Attribution License 4.0).

T cells are the adaptive immune system component that initiates the production of interleukins such as IL-4, 10, and 17, along with IFN-γ. The Mucorales hyphal encounter triggers the immune regulation of these interleukins by generating Mucorales-specific T cells through stimulation of CD4+ T cells (Castillo et al., 2018). Another category of cells named NK cells is involved in pathogen clearance by reducing dissemination (Vivier et al., 2008). In a study, it is found that Mucorales hyphae are destroyed by stimulated and unstimulated NK cells except resting (dormant) spores mediated by perforin protein secreted by the NK cells (Schmidt et al., 2016). Similarly, platelets and endothelial cells play an important role in the recognition and killing of Mucorales hyphae and phagocytosis of the fungal spores (mediated by glucose-regulated protein 78), respectively (Liu et al., 2010; Jenne and Kubes, 2015). Mucorales interaction with the host immune system is modulated by several factors, including hyperglycemia and DKA. Deferoxamine- and DKA-treated patients are shown to be predisposed to mucormycosis. Diabetic subjects show uncontrolled hyperglycemia, increased ketone bodies [e.g., β-hydroxy butyrate (BHB)] and low pH due to accumulation of these ketone bodies in the blood that impairs the transferrin protein binding characteristic to chelate iron (Figure 3). These factors, including high blood glucose, excessive iron, and the presence of BHB promote the growth of fungus mediated by suppression of T-lymphocyte induction as shown in Figure 3.

## Immunomodulatory Protective Mechanism of Mscs Prospectively in Mucormycosis

**Figure 4:**
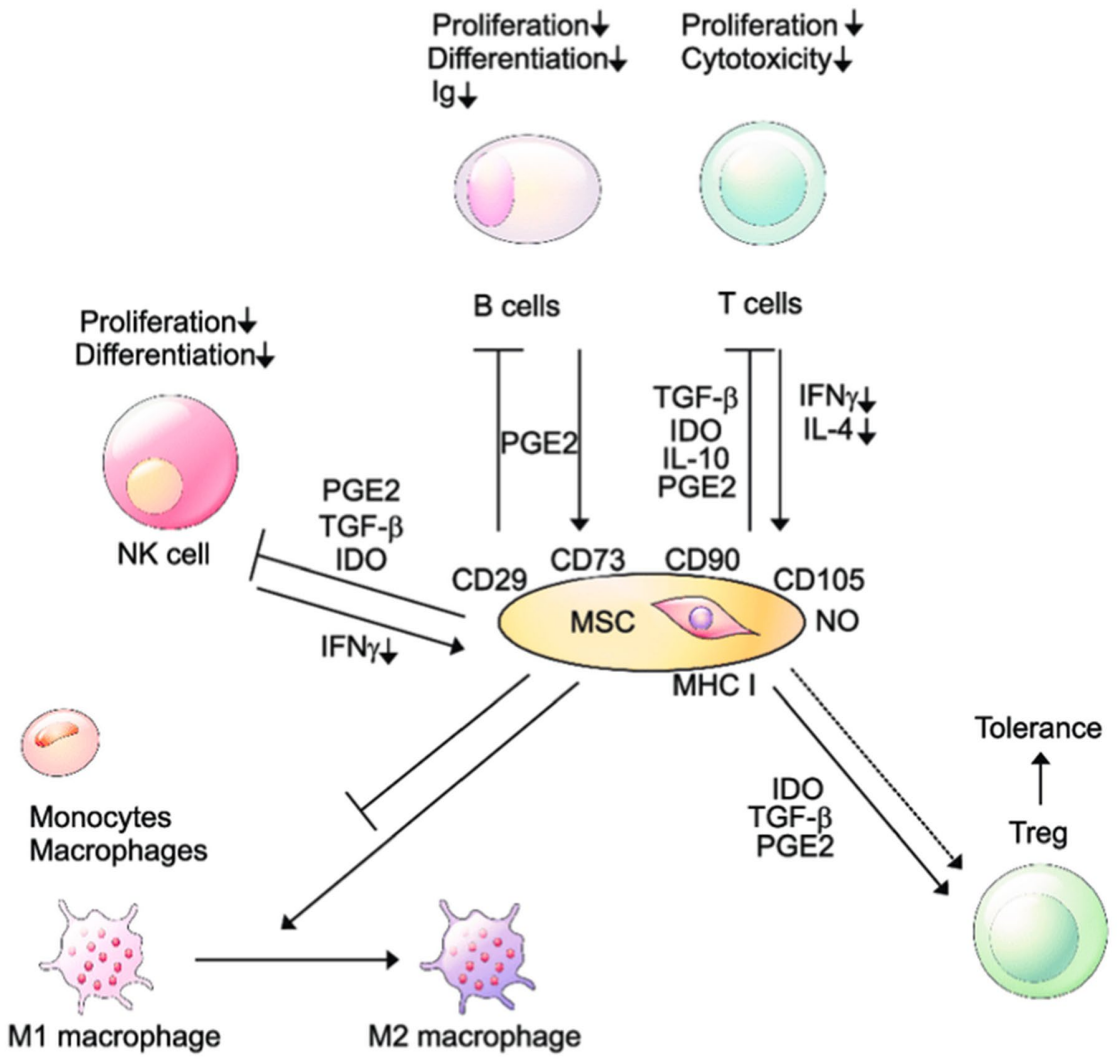
Immunomodulation by MSCs. Ig, immunoglobulin; NK, natural killer; PGE, prostaglandin E; TGF, transforming growth factor; IDO, indoleamine 2,3-dioxygenase; IFN, interferon; IL, interleukin; MHC, major histocompatibility complex, NO, nitric oxide. (Adapted from Ref (Kang et al., 2020) under the terms of the Creative Commons Attribution License 4.0).

The mechanism by which MSCs exert immunomodulation in host cells is mediated by inhibition of CD4+ and CD8+ T cells, B lymphocytes, and NK cells. Human bone marrow–derived stem cells (hBMSCs) exhibit CD73+, CD90+, and CD105+ along with CD45-, CD34-, CD14-, CD11b-, CD79a-, and HLA class II (Dominici et al., 2006; Ma et al., 2014; Figure 4). Studies show that hBMSCs produce several immunomodulatory factors, including chemokines, cytokines, growth factors, and proteins of extracellular matrix that play a significant role in building immunity of the host and reducing the inflammatory response along with tissue regeneration (Burrello et al., 2016). hBMSCs recognize the invading pathogen by TLR present on the cell surface and express antimicrobial peptides; in case of antigenic presentation, these cells may behave as pro-inflammatory and immunosuppressive cells (Auletta et al., 2012).

In recent research conducted on a murine model of chronic pulmonary paracoccidioidomycosis, BMSCs show a promising approach in improving health outcomes and boosting immune responses mediated by depletion of the neutrophils (Puerta-Arias et al., 2016). *In vitro* and *in vivo* study demonstrates that IL-17+ MSCs inhibit the growth of *Candida albicans*. MSCs are proven to show a protective effect in asthma induced by *Aspergillus fumigatus* hyphal extract mediated by inhibition of Th17 signaling mechanism (Yang et al., 2013). Similarly, BMSCs induce an immunomodulatory effect on *A. fumigatus* conidia-stimulated macrophages through step-down of TNF-α secretion and step-up of IL-10 production (Lathrop et al., 2014).

Mucorales triggers the immune response postrecognition by the endothelial cells on an antagonistic counterpart, and MSCs modulate and reverse these immune responses and protect the host from infection such as in mucormycosis by inducing naïve and effector T cells, suppression of NK cells, increased IL-17 secretion, neutropenia, and inhibiting the cytokine storm at the site of fungal infection. MSCs, apart from inducing immunomodulatory characteristics, regenerate the infected skin/tissue, which an antifungal drug is unable to do. In a previously published study, the antifungal host response effect of human MSCs (hMSCs) was investigated on anti-Aspergillus CD4+ T cells, and it was found that anti-Aspergillus T cells initiate the IL-6 production of hMSCs (Schmidt et al., 2017).

MSCs modulate the immune molecules of the immune system and are proven to be beneficial in the survival of patients suffering from immune-related complications. MSCs are known to prevent the overstimulation of the immune system as they contribute to the suppression of the immune functions mediated by stimulation of pro-inflammatory factors (IFN-γ, TNF-α, and IL-1β; Jiang and Xu, 2020; Table 1). Among these immune modulators, IFN-γ plays a crucial role in controlling the suppressive function of MSCs as it stimulates the expression of programmed cell death ligands 1 and 2 (PD-L1/L2) along with downregulation of immunoglobulin-like transcript receptors (ILTRs; Jiang and Xu, 2020). It is supported by other literature, in which the authors give Nivolumab along with IFN-γ and find promising results in the treatment of intractable mucormycosis (Grimaldi et al., 2017). Furthermore, immunosuppressive characteristics of MSCs also contribute in downregulating the expression of MHC-I, MHC-II, and FasL along with CD80, CD86, CD40, and CD40L factors (Jiang and Xu, 2020). The in-built property of the MSCs to express chemokines along with several adhesion proteins help in the recruitment of immune cells such as C-X-C motif chemokine receptor 3 (CXCR3), C-C motif chemokine receptor 5 (CCR5), vascular cell adhesion molecule 1 (VCAM-1), and intercellular adhesion molecule 1 (ICAM-1) ligands that also help in the suppression of inflammatory mechanisms (Jiang and Xu, 2020). Recently, published studies demonstrate the role of small molecules, including monoclonal antibodies, vaccines, peptides, and interferon in the treatment of the SARS-CoV-2 that might be explored further in treatment of SARS-CoV-2–associated mucormycosis.

**Table 1:** Immunological modulation of MSCs.

Immunomodulatory factors	Species	Roles in MSC-mediated immunosuppression	References
iNOS	Murine MSCs	Inhibits T-cell proliferation	Ren et al., 2008
CCL2	Murine MSCs	Inhibits CD4+ Th17 cells	Rafei et al., 2009
IDO	Human MSCs	Inhibits T-cell proliferation	Ren et al., 2009
Semaphorin-3A	Human MSCs	Inhibits T-cell proliferation	Lepelletier et al., 2010
B7-H4	Human MSCs	Inhibits T-cell activation and proliferation	Xue et al., 2010
HLA-G	Human MSCs	Inhibits PBMC response	Rizzo et al., 2008
Galectin(s)	Human MSCs	Inhibits T-cell proliferation	Lepelletier et al., 2010
HO-1	Murine MSCs	Inhibits T-cell response	Chabannes et al., 2007
IL-6	Murine MSCs, human MSCs	Inhibit the differentiation of dendritic cells; inhibit T-cell proliferation	Djouad et al., 2007; Najar et al., 2009
TGF-β	human MSCs	inhibits NK cell activation and function	Sotiropoulou et al., 2006
IL-10	Murine MSCs, human MSCs	Inhibits T-cell responses, decreases Th17 cell differentiation	Rasmusson et al., 2005; Qu et al., 2012
PGE2	Murine MSCs,	Induces Foxp3+ Tregs	English et al., 2009
FasL	human MSCs	Induces T-cell apoptosis	Akiyama et al., 2012

*CCL2, chemokine ligand 2; DC, dendritic cells; FasL, Fas ligand; HLA-G, human leukocyte antigen G; HO-1, heme oxygenase-1; IDO, indoleamine 2,3-dioxygenase; iNOS, inducible nitric oxide synthase; LIF, leukemia inhibitory factor; MSCs, mesenchymal stem cells; PGE2, prostaglandin E2; PD-L1/2, programmed cell death 1 ligand1/2; PBMC, peripheral blood mononuclear cells; TSG6, TNF-α stimulated gene/protein 6. [Adapted from Ref (Rafei et al., 2009) under the terms of the Creative Commons Attribution License 4.0]*.

## Conclusion

Invasive mold infections vary from patient to patient depending upon the immunocompromised ability of the immune system in the infected host. Recently, mucormycosis is showing an increasing trend due to several reasons associated with COVID-19, including comorbidities such as DM, DKA, prolonged steroid treatment, that hamper SARS-CoV-2-infected host immunity and provide an opportunity for Mucorales to inhabit the host. Early diagnosis of these species should be a matter of concern by using molecular biological assessment tools along with the clinical diagnosis. Because antifungal drugs (itraconazole, posaconazole, or voriconazole) having the azole functional group is the first choice in mucormycosis possess some limitation of having side effects on long-term use, MSCs can be an alternative cell-based treatment approach that can be used without conflict. MSCs exhibit an immunomodulatory role in the host and protect it from fungal infection by boosting the immune system through the release of various chemotactic proteins.

Moreover, very limited studies are present explaining the mechanism of immunomodulation adopted by MSCs in mucormycosis. However, assumptions can be made for using MSCs as prospective therapy in the treatment of mucormycosis. No conclusive data of clinical trials are available to include MSCs in treatment of mucormycosis.

## Author’s note

Currently involved in COVID-19 testing duties; Prashant Tripathi: Nodal Officer, COVID-19 Testing Laboratory, GSVM Medical College, Kanpur; Richa Giri: Nodal Officer, Multidisciplinary Research Unit, GSVM Medical College, Kanpur.

## Author Contributions

AR, SA, and G-BJ: conceptualization. AR, SA, G-BJ, KG, SB, QM, SR, and RT: formal analysis. KG, SB, SR, and RT: investigation. AR, SA, and G-BJ: methodology. PT, RG, SA, MS, and HK: supervision. PT, RG, SA, MS, and HK: visualization. AR, SA, G-BJ, and QM: writing – original draft. PT, RG, SA, MS, and HK: writing – review and editing. All authors have read and approved the manuscript and ensure that this is the case.

## Conflict of Interest

The authors declare that the research was conducted in the absence of any commercial or financial relationships that could be construed as a potential conflict of interest.

## Publisher’s Note

All claims expressed in this article are solely those of the authors and do not necessarily represent those of their affiliated organizations, or those of the publisher, the editors and the reviewers. Any product that may be evaluated in this article, or claim that may be made by its manufacturer, is not guaranteed or endorsed by the publisher.

## Abbreviations

SARS-CoV-2: Severe Acute Respiratory Syndrome Coronavirus 2
MSCs: Mesenchymal Stromal Cells
COVID-19: Coronavirus Disease-19
CD: Complementary Determining
ISCT: International Society of Cellular Therapy
NK cells: Natural Killer Cells
CNS: Central Nervous System
USA: United States of America
FDA: Food and Drug Administration
HSC: Hematopoietic Stem Cells
IL-6: Interleukin −6
DM: Diabetes Mellitus
DKA: Diabetic Ketoacidosis
ROCM: Rhino-Orbital-Cerebral Mucormycosis
TLR-2: Toll-Like Receptors 2
CT: Computerized Tomography
RHS: Reverse Halo Sign
PET/CT: Positron Emission Tomography-Computed Tomography
FDG: fluorodeoxyglucose
RFLP: Restriction Fragment Length Polymorphism Analyses
MALDI-TOF MS: Matrix-Assisted Laser Desorption Ionization-Time of Flight Mass Spectrometry
muMSCs: Murine Mesenchymal Stem Cells
AML: Acute Myeloid Leukemia
TNF-α: Tumor Necrosis Factor-α
BAM: Bronchial Alveolar Macrophages
PDGF RB: plaTelet-Derived Growth Factor Receptor B
PMNs: Polymorphonuclear Leukocytes
hBMSCs: Human Bone Marrow Derived Stem Cells
